# Mobile phone radiation health risk controversy: the reliability and sufficiency of science behind the safety standards

**DOI:** 10.1186/1478-4505-8-2

**Published:** 2010-01-27

**Authors:** Dariusz Leszczynski, Zhengping Xu

**Affiliations:** 1 STUK - Radiation and Nuclear Safety Authority, Laippatie 4, FIN-00880 Helsinki, Finland; 2 Zhejiang University, School of Medicine, 388 Yu-Hang-Tang Rd, Hangzhou 310058, China

## Abstract

There is ongoing discussion whether the mobile phone radiation causes any health effects. The International Commission on Non-Ionizing Radiation Protection, the International Committee on Electromagnetic Safety and the World Health Organization are assuring that there is no proven health risk and that the present safety limits protect all mobile phone users. However, based on the available scientific evidence, the situation is not as clear. The majority of the evidence comes from *in vitro *laboratory studies and is of very limited use for determining health risk. Animal toxicology studies are inadequate because it is not possible to "overdose" microwave radiation, as it is done with chemical agents, due to simultaneous induction of heating side-effects. There is a lack of human volunteer studies that would, in unbiased way, demonstrate whether human body responds at all to mobile phone radiation. Finally, the epidemiological evidence is insufficient due to, among others, selection and misclassification bias and the low sensitivity of this approach in detection of health risk within the population. This indicates that the presently available scientific evidence is insufficient to prove reliability of the current safety standards. Therefore, we recommend to use precaution when dealing with mobile phones and, whenever possible and feasible, to limit body exposure to this radiation. Continuation of the research on mobile phone radiation effects is needed in order to improve the basis and the reliability of the safety standards.

## Introduction

There is continuously ongoing controversy whether the users of mobile phones should be concerned about:

(i) the health safety of the radiation emitted by these devices,

(ii) whether the safety standards are adequate and,

(iii) whether continuation of research in this area is scientifically justified.

The International Commission on Non-Ionizing Radiation Protection (ICNIRP), the International Committee on Electromagnetic Safety (ICES) and the World Health Organization Electromagnetic Fields Project (WHO EMF-Project) are assuring users that there is no proven health risk and that the present safety standards on the radiation emitted by mobile phones protect all users [1-3]. However, because of the methodological and research design limitations that are intrinsic to different types of studies (epidemiology, human volunteers, animal and *in vitro *studies) the so far gathered scientific evidence is, in our opinion, still insufficient to support these claims.

When evaluating the possible health effects of mobile phone radiation, as with any other environmental factor, no matter naturally occurring or man-made, are needed several types of scientific evidence such as (i) the possible mechanism how the effect is induced in living organism, (ii) *in vitro *laboratory studies that confirm the existence of a biophysical and biochemical mechanism of the effect, (iii) animal studies, (iv) human volunteer studies, and (v) epidemiological evidence of the effect on human population. Each type of the evidence is of different significance and value for the estimation and proof of human health effect. The most important is epidemiological evidence, followed by the human volunteer studies and animal experiments. *In vitro *evidence does not directly inform about the possible health impact but it provides information about the possible mechanism of the effect on cellular level. Knowing the mechanism of the effect increases the reliability of the evidence gathered in epidemiological, human volunteer, animal and *in vitro *studies. In the ideal situation, all above listed types of scientific evidence would point into the same direction.

This is, however, not always the case. For example electromagnetic fields emitted by the power-lines (ELF-EMF) were classified by the International Agency for Research on Cancer (IARC) as a possible carcinogen (category 2B), based predominantly on the epidemiological evidence because there was no clearly supporting evidence from the animal and *in vitro *studies [4]. However, recently published study provides new evidence suggesting that ELF-EMF might interfere with the DNA repair process what might cause accumulation of damaged DNA in cells [5]. If so, such process could be considered as at least potential trigger for the development of cancer. In the context of this new methodological approach to the issue of ELF-EMF-induced DNA damage, calls for ceasing further research in area of ELF-EMF and cancer [6] or similar calls for cease of research on mobile phones [7] are certainly premature. This new study [5] also underlines the need for revisiting of old, unsolved issues, by using novel experimental techniques and methodological approaches to get a better insight into possible biological effects of power lines and mobile phones.

The evidence concerning biological and health effects of mobile phone radiation is contradictory. In each area of investigation (epidemiology, human volunteer studies, animal and *in vitro *studies) there are both studies showing effects and studies showing no effects of mobile phone radiation. When considering just the sheer numbers of published studies, the number of the no-effect studies is larger than the number of the studies that show an effect. This is commonly referred as the "weight of the evidence" that is pointing out to the no-effect-result, as the prevailing one. This argument is often used as evidence indicating that there are no health effects below the present safety standard limits [1-3].

However, when evaluating reliability of the present safety standards we need to consider not only the already available scientific evidence. We also need to consider the "missing" evidence. It means that we need to determine what kind of important health-related studies have not yet been done and, therefore, what important health-related evidence we are missing. Only when considering both, the available and the missing evidence, we can more reliably judge the reliability of the current safety standards.

The vast majority of the research studies examining biological and health effects of mobile phone radiation have focused on the possibility of induction of cancer. At the same time, the discussion continues on whether mobile phone radiation could cause effects that, although not able to develop into life-threatening disease, could become detrimental to the quality of life. These life non-threatening effects might include such ailments as e.g. sleep disorders, headaches or allergy-like symptoms. Therefore, independently of their outcome, the majority of the to date executed studies provides information about the cancer and are unable to give mobile phone radiation "a clean bill of health" by showing that the exposure to this radiation is not associated with any other, non-cancer-related, health risk.

## Epidemiological studies

Epidemiological studies are considered as the most important in evaluation of human health risk. However, due to their low sensitivity in detecting health effects within the population, epidemiology alone is unlikely to be able to conclusively determine whether weak stimulus, such as mobile phone radiation, causes cancer or other ailment. Besides the low sensitivity of this method, there are, as reviewed by Kundi [8], numerous biases that affect health risk estimation by epidemiology, such as: selection bias, misclassification bias, recall bias, and the effect of the developing disease on mobile phone use. Furthermore, as reviewed by Kundi [8] there are methodological considerations in epidemiological studies that are unsolved at the moment, such as: no evidence based exposure metric, low duration of mobile phone use, and no evidence-based selection of end-points for epidemiological studies.

Further complication with the epidemiological evidence is the long latency period (over 10 years) between the induction and diagnosis of brain cancer. Therefore, it is not surprising that the majority of the to date executed epidemiological studies, covering at the longest the period of the first 10-years after the start of use of mobile phone, do not show any link between brain cancer and mobile phone radiation [8]. However, even if such causal link would exist, it might be not detectable in such short-term studies because the numbers of phone users 10-20 years ago were small.

Great hopes for the answers whether there is a causal link between brain cancer and mobile phone radiation exposure were with the EU 5^th ^Framework Programme-funded "Interphone Project" [9]. This project is a multinational case-control study that was set-up to investigate whether mobile phone radiation increases the risk of brain cancer. The hallmark of this project was supposed to be the common core protocol that would allow to pool data obtained in all 13 participating countries and to perform a combined statistical analysis. Such combined analysis would then consist of a sufficient number of cases and controls to be statistically meaningful and informative [9]. However, as it appears there were numerous exceptions from the common core protocol [9] and they might have bearing on the outcome of the final combined analysis.

Another problematic issue of the Interphone project is the definition of a regular user of the mobile phone. As the regular user it is defined a person who makes at least one phone call per week for the period of at least 6 months. This means that person who makes total of 24-25 calls over 6 months period is analyzed in the same category of regular user as a person who makes 24-25 calls per day. This means that the category of regular users consists of those who are very little exposed to mobile phone radiation and of those who are very heavily exposed. It may mean that, if we assume that the heavy exposure to mobile phone radiation could cause any health effects, the average effects of exposure might be diminished when analyzing in the same group the very low- and the very high-exposed study subjects.

Therefore, when using Interphone's definition of "regular user" that might "dilute" the effects, it is somewhat surprising that there are Interphone studies suggesting the increase in brain cancer among the long-term "regular users" (>10-years) [10,11].

Standing out among the epidemiological studies is a series of papers published by the group of Lennart Hardell from Sweden (for review see [12]). These studies show that the exposure to mobile phone radiation causes increase in risk of brain cancer. There is ongoing discussion why the results obtained by Hardell and co-workers differ from the results obtained in other studies. The reasons for such discrepancy remain still elusive. In the recently published review of epidemiological studies by the ICNIRP's Committee on Epidemiology the authors have concluded that they do not know what might be the cause of this discrepancy [13]. Because of it, the combined analysis of epidemiological studies was presented in this ICNIRP's review in two formats: (i) results of all studies together and (ii) results of all studies but without Hardell studies. The first analysis has shown a possibility of a small increase in cancer risk whereas the second analysis has shown no risk at all. In the conclusions, the authors of ICNIRP's review have leaned towards the no-risk-effect as the summary outcome of all so far executed epidemiological studies. Reliability of this conclusion was, however, questioned in the accompanying editorial article [14], where Ken Rothman has stated that "*Skeptics might rightly take this as only mild reassurance, because induction times for radiation caused tumors often exceed 10 years*."

The ICNIRP analysis is in clear contract with the recent meta-analysis of epidemiological studies performed by researchers from South Korea and USA [15]. Interestingly, in this meta-analysis the studies published by Hardell and co-workers were considered as of higher quality than many other studies. This is on the contrary to the ICNIRP review. Consequently, the conclusion of the meta-analysis was that "*The current study found that there is possible evidence linking mobile phone use to an increased risk of tumors from a meta-analysis of low-biased case-control studies*".

Thus, there seems to be a major discrepancy not only in the results of the executed case-control epidemiological studies but also in deciding which of the studies are methodologically of better, more reliable, quality.

However, apart of the variations in analyses of epidemiological studies by different groups of scientists, the major problems with the current epidemiological evidence are the intrinsic methodological limitations of case-control studies. Anssi Auvinen has stated at the joint meeting of Bioelectromagnetics Society and the European Bioelectromagnetic Association (Davos, Switzerland, 2009) that, we have performed sufficiently many epidemiological case-control studies but their results are of insufficient quality to reliably draw any health-risk-related estimates.

*Summa summarum*, it appears that in order to get more reliable answers from epidemiologists, we need to wait for the outcome of Cosmos cohort study that has just started in Finland, Sweden, Denmark, Holland and Great Britain.

## Human volunteer studies

The human volunteer studies have focused on mobile phone radiation effects on e.g. cognition, blood pressure, headaches, skin allergy-like symptoms, sleep disorders or direct recognition, by the exposed subject, whether mobile phone emits radiation or is switched off [16]. These studies have one major set-back - experimental environment and used exposure and measurement hardware can psychologically affect behavior of the volunteers during the experiments and the obtained information might become subjective and unreliable [16].

Therefore, in addition to such studies we also need studies that would examine whether human body responds to mobile phone radiation on molecular level. It is certain that just such human volunteer studies, using methods of proteomics, transcriptomics and other reliable biochemical analyses, are urgently needed to demonstrate whether human body (tissues, organs) responds, or not, to mobile phone radiation. Such studies will not only show whether human body recognizes mobile phone radiation as an external stressor but also will provide information which molecules, proteins and genes react to mobile phone radiation. With this information in hand it will be possible to formulate new, knowledge-based, hypotheses for further health risk related studies in humans [17,18].

As stated already, to this time, we do not have available objective information whether human body recognizes mobile phone radiation (at levels permitted by the current safety standards) as an external stressor and responds to it at molecular level. Such responses are prerequisite for any physiological/health-related responses. Therefore, because of the lack of studies that would provide unbiased information whether the human body responds to mobile phone radiation, it is problematic to consider that the presently available safety standards protect all users of mobile phones [19]. Such claims might be premature in situation when we still do not know whether human body reacts to mobile phone radiation at all.

The effects of mobile phone radiation on children and recommended prudent use of mobile phones are one of the more discussed issues [20-23]. The present safety limits are considered to protect also children [24]. However, in part due to ethical considerations, there are no published studies where the effects of mobile phone radiation on development or health of children would have been examined. The scientific evidence comes only from the studies examining young animals and its applicability to human children might be of limited value.

The presently used safety standards might very well protect the majority of mobile phone users. However, there likely exists a subpopulation of people with different sensitivity to mobile phone radiation (not to confuse with the self-diagnosed so-called Electromagnetic Hyper Sensitivity - EHS). It is known that due to genetic variability among people, the same physical or chemical agents (medication, radiation, chemicals, allergens, etc) may elicit responses of differing severity in different people [25] - the so-called individual sensitivity. Finding out such sensitive subpopulation and defining it might be only possible by examining molecular level responses to this radiation [18].

## Animal and *in vitro *studies

Animal studies are commonly used when examining whether physical and chemical agents affect human health. In *sensu stricto *toxicology studies, animals are treated with a large overdose of tested agent, which would not be encountered by human being in real life situation. However, such toxicology studies are not possible to perform for mobile phone radiation (microwaves). Overdose of microwave radiation, above the level of current safety standards, will heat up the animal and, in extreme cases it might simply "cook it". Interpretation of such studies, in respect to human health risk, might be very difficult because of the temperature increase of the animal. The current safety standards are set specifically to protect from such thermal effects of mobile phone radiation.

The other kind of animal studies are these where animals were exposed for different periods of time to doses of mobile phone radiation that are permitted by the current safety standards and that do not cause heating of the animal. However, direct extrapolation of the results of such animal studies, performed at low doses of mobile phone radiation, to human health risk is also problematic. It is known that although humans and animals possess many of the same genes, the functions of the same genes might differ and some of the same cancer types are regulated by different genes in animals and in humans. This causes that some of the cancers that will appear in animal will not appear in humans and vice-versa [26]. Also, lack of the effects of low-dose exposures in animals does not automatically mean that this will apply also to humans.

The majority of research on the biological effects of mobile phone radiation has been done in laboratory *in vitro *studies and the vast majority of the conducted research has focused on cancer. Some of the *in vitro *studies suggest, although do not prove, that mobile phone radiation might alter cell physiology e.g. by triggering cellular stress response, causing DNA damage, altering gene and protein expression [20-22]. However, there are also numerous studies that do not see such effects [20-22].

One of the more vigorously debated issues is whether mobile phone radiation is able to cause DNA damage. There have been performed numerous studies on this subject and while the majority of them shows no effect, there are some studies suggesting that the DNA might be damaged by mobile phone radiation. However, nearly all studies have used the same methodological approach - the comet assay, micronuclei formation and chromosomal aberrations. It seems clear that without changing the methodological approach, the issue of DNA damage might not be reliably resolved in the near future. This call for changing methodological approach applies also to other areas of EMF research because when looking at the EMF publications one gets impression that this research is "stuck" on replication and re-replication of the studies using the same, often outdated, methods that in the end do not give resolution to the problem.

Finally, even though there have been executed numerous *in vitro *laboratory studies, these *in vitro *studies are of value only for discovering the biochemical mechanism of the effect and they provide support for human and animal studies, but they can not be directly used to determine the probability of health risk or in providing information for setting of human health safety standard.

## Conclusions

We still are missing some of the basic information that is required to determine whether mobile phone radiation could be hazardous for humans and whether our safety standards are adequate - we do not know whether human body reacts at all to mobile phone radiation. If the answer is yes then: are children more sensitive than adults and what could be the consequences of prolonged exposures to this radiation, when users will be exposed for their life-time.

In the absence of such information, any statements indicating that the use of mobile phones has been shown to be safe might be premature. After all, we also need to remember that the mobile phone radiation is not a natural part of human environment and evolution did not prepare our bodies for such exposure. The last 10-20 years are the first years in the history of human species when our brains are being closely and directly exposed to this, novel to them, radiation. The current safety standards might be the best what can be done using the presently available scientific evidence and they should not be altered arbitrarily, without scientific justification. However, these standards are not yet sufficiently supported by the science and can not be considered as scientifically reliable.

This is why we should continue research in this area. The reason for continuation of research is not just science for the science's sake. The reason is that our scientific evidence is insufficient to support the notions that there will be no health effects and that the safety standards are sufficient to protect all users. The present situation of scientific uncertainty calls for both precautionary measures and for further research.

## Abbreviations

ICNIRP: International Commission on Non-Ionizing Radiation Protection; ICES: International Committee on Electromagnetic Safety; WHO EMF Project: Electromagnetic Fields Project of the World Health Organization; IARC: International Agency for Research on Cancer.

## Competing interests

The authors declare that they have no competing interests.

## Authors' contributions

DL has prepared the first version of the manuscript. DL and ZX have equally participated in revising and modifying the manuscript to its final version.
